# The interplay between endorser social status and normative appeals on the endorsement effectiveness of pro-environmental behaviors

**DOI:** 10.1371/journal.pone.0210699

**Published:** 2019-01-15

**Authors:** Hao He, Jingtao Fu, Xi Li, Rui Guo

**Affiliations:** 1 School of Economics and Management, Changsha University of Science and Technology, Changsha, Hunan, China; 2 School of Economics and Management, Hainan University, Haikou, Hainan, China; 3 Division of Business and Management, Beijing Normal University-Hong Kong Baptist University United International College, Zhuhai, Guangdong, China; 4 School of Economics and Management, China University of Geosciences, Wuhan, Hubei, China; Central South University, CHINA

## Abstract

Employing message endorser is a popular strategy in encouraging consumers to protect the environment. This research explores how the social status of endorsers and the forms of normative messages can influence the effectiveness of endorsement for pro-environmental behaviors. Drawing on the focus theory of normative conduct and the match-up hypothesis, the authors propose that the effects of endorser social status on consumers’ responses to green advertising are contingent on whether the normative messages is framed as injunctive norms or descriptive norms. In three experiments, the results indicate that participants show more positive attitudes toward the advertisement and higher intentions to act environmentally friendly when endorsers with high social status are presented in combination with injunctive norm appeals. In contrast, ordinary consumer endorsers produce stronger impact on attitudes and behavioral intentions when descriptive norm appeals are used. These findings show that marketers using endorsers to promote pro-environmental behaviors should develop normative message accordingly.

## Introduction

Though substantial research effort has been put into the political and industrial level of environment protection [1], the negative impact of consumption behaviors on the environment can not be underestimated. Previous studies have pointed out that daily consumption activities are critical contributors to environmental deterioration [2], and as a result, adopting appropriate strategies to foster pro-environmental behaviors among consumers is now becoming an important issue for governments, social marketing practitioners and scholars all over the world [3].

For green advertising practices, we can often see celebrities as well as ordinary consumers as endorsers in pro-environmental advertisements in an effort to encourage consumers to behave in an environmentally friendly manner. For example, the World Wildlife Fund (WWF) in China not only invited Li Bingbing, a famous Chinese actress, to be its spokesperson, but also used the image of a primary school student named Wang Ruihui to promote energy conservation practices. Questions can be raised such as the following: Are there any differences in the endorsement effectiveness between these different kinds of endorsers in encouraging consumers’ intentions to protect the environment? Should marketing practitioners develop the same or different message formulation for each type of endorsers?

In the context of commercial advertising, endorsement effectiveness has been examined along several dimensions. For example, endorsers perceived to be highly attractive, trustworthy and with great expertise are found to be more effective in helping consumers process product-related information [4]. However, the role that social status can play in endorsement effectiveness has received limited attention [5] despite the fact that some endorsers (e.g., celebrities) enjoy higher social status than do the others (e.g., ordinary consumers) [6]. Social status is the relative standing a person holds within a social hierarchy [5, 7]. Higher status individuals have greater access to desirable benefits, such as resources, social approval, and respect from others [5, 8]. As previous literature shows, status concerns play a key role in motivating green consumption [8] and the symbolic meaning of endorser’s status can be transferred to the endorsed activities [9], we believe that it is of great importance to ascertain how the social status of endorsers would affect consumers’ reactions to pro-environmental appeals. Therefore, the current research seeks to advance prior research by examining the interplay between endorser social status and normative appeals on endorsement effectiveness in the context of pro-environmental advertising.

Using the power of social norms in green advertising is an important way to promote pro-environmental behaviors [10]. Previous research differentiates two forms of normative information: injunctive norms (perceived degree of social approval/disapproval of a given behavior), and descriptive norms (perceived prevalence of a given behavior) [11]. Both forms of normative information influence pro-environmental behaviors, yet each represents a distinct source of motivation [10, 11]. Whereas injunctive norms are associated with goals to gain social approval, descriptive norms are relevant to goals to make adaptive decisions [12]. Based on the match-up hypothesis, which stresses the fit between the endorsers and the endorsed offerings [13], we propose that the effectiveness of endorsers of pro-environmental behaviors with different social statuses will depend on the focus of normative appeals. That is, when the social status of a pro-environmental behavior endorser is congruent with the goals to follow different types of social norms, the endorsement effectiveness will be enhanced. Therefore, this research represents a preliminary exploration of the question as to how to boost the persuasiveness of pro-environmental advertisements by combining endorsers and normative appeals, and provides theoretical guidance for governments and environmental nonprofit organizations to design more effective advertising.

This article is structured as follows. First, we review literature about endorsement effectiveness and normative appeals to provide a theoretical basis for our hypotheses. After that, we examine the hypotheses with 3 experiments using different ways to manipulate endorser social status. At last, we make some discussions about our findings and provide suggestions for future research.

## Literature review and hypotheses

### Effectiveness of endorsement

Endorsement is a popular advertising strategy to promote the adoption of products or behaviors [4, 5, 14]. Existing research in endorsement mainly adopts the source-credibility theory [4, 15] and the match-up hypothesis [14, 16] to explain endorsement effectiveness, which is defined as the extent to which an endorser exerts influences in improving advertising attitudes and purchase intent [17]. According to the source-credibility theory, an endorser’s credibility, which comprises trustworthiness, expertise, and physical attractiveness, plays an important role in the advertising message’s effectiveness [4, 18, 19]. Due to their wide recognition and popularity, many studies find celebrity endorsers to be more credible and effective than noncelebrity endorsers are [17, 18].

Meanwhile, as some research suggests that the effectiveness of celebrity endorsers is contingent on the product being endorsed [16, 20], the match-up hypothesis highlighting the fit between an endorser and the endorsed product is then proposed to explain endorser effectiveness [13, 16, 17]. Existing studies examine the match-up hypothesis in numerous contexts and identify several factors that lead to a good match-up effect. For example, physically attractive celebrities are more effective for products related to attractiveness [16, 20, 21]; expert endorsers are more persuasive than celebrity endorsers for high technology products [22]; and celebrity endorsers are more appropriate than noncelebrity endorsers for products with a high social or psychological risk [23], or products deemed to be symbols of social status [24].

While the existing literature provides fruitful insights for a thorough understanding of endorsement effects in commercial advertising contexts, ways to design an effective endorsement campaign to promote pro-environmental behaviors are overlooked. Previous literature demonstrates that pro-environmental behaviors can be a signal of social status because of the associated self-sacrifice to benefit the societal collective well-being [8, 25]. Following the rationale of the match-up hypothesis, an endorser with high social status (e.g., a celebrity) is assumed to be more effective than a common individual (e.g., an ordinary consumer) is for encouraging consumers to behave in an environmentally friendly manner. However, the status-displaying effect of pro-environmental behaviors is attenuated when the relevant costs are reduced [8], which suggests that a prevalent pro-environmental behavior may undermine the effectiveness of high-status endorsers. Thus, a careful examination of match-up factors in the pro-environmental context is still necessary. In the current research, we try to fill this gap by exploring how the match-up between social status of endorsers and focus of normative appeals will influence the effectiveness of pro-environmental endorsement.

### Normative appeals

Social norms have proven to be influential determinants of pro-environmental behaviors [10, 11, 26]. The focus theory of normative conduct discriminates between injunctive norms and descriptive norms [11]. The injunctive norms refer to the perceived degree that a given behavior is socially approved and disapproved. In contrast, the descriptive norms describe the typical or normal conduct that most people perform [11]. For example, trash-sorting behavior can be framed as “everyone should do trash-sorting” (injunctive norm) or as “many people around you have done trash-sorting” (descriptive norm). Existing research suggests that both injunctive norms and descriptive norms can affect pro-environmental behaviors of consumers [10, 11]. For instance, people are less likely to litter in a heavily littered environment with a competing injunctive norm against littering, or in a previously clean environment (i.e., descriptive norm suggesting most people not littering) [11].

The focus theory of normative conduct indicates that people are motivated to conform to these two types of normative appeals with fundamentally different goals. Whereas the injunctive norm-consistent actions help to fulfill an interpersonal goal of acquiring social approval, the descriptive norm-consistent actions pertain to an intrapersonal goal of behaving effectively and adaptively [11, 12]. Previous research provides some evidence for this distinction of goal-directed influence between the injunctive norm and the descriptive norm. For example, Jacobson, Mortensen, and Cialdini [12] found that participants primed with words related to injunctive norms (e.g., ought, responsibility) reacted faster to words associated with social approval goals (e.g., approve, team) than did those primed with words related to descriptive norms (e.g., typical, popular); the response to words relevant to accuracy/efficiency goals (e.g., accurate, beneficial) was slower when participants were primed with injunctive-norm words than when primed with descriptive-norm words. They also found that exposure to an injunctive norm made interpersonal goals more salient than exposure to a descriptive norm. White and Simpson [10] provided further evidence for this goal difference in a context of grasscycling. They found that when the individual level of self was activated, the descriptive norm message produced better persuasive effects than those of the injunctive norm message. When the collective level of self was activated, consumers responded similarly to the injunctive and descriptive norm information [10].

### Effects of normative appeals on pro-environmental behavior endorsement

Unlike conventional product-purchasing and health behaviors, which are directly relevant for consumers’ own personal outcomes [5], pro-environmental behaviors lead consumers to face a trade-off between costs to the individual-self (e.g., inconvenience, increased effort, behavioral change) and benefits to the societal collective well-being [10, 27]. According to the focus theory of normative conduct, injunctive norms and descriptive norms represent distinct goals for solving such trade-off involved in decisions to adopt pro-environmental behaviors [10]. Injunctive norms are followed with goals to seek social approval [11,12], and consumers may try to differentiate themselves positively from others (i.e., acquiring higher social status) by fulfilling social obligations (e.g., protect the environment) associated with costly self-sacrifice [8, 25]. In contrast, consumers who follow descriptive norms may try to engage social obligations in an efficient way (e.g., with a minimal level of cost to self-interest) and appear socially adaptive by observing and imitating what others are doing [11, 28].

As the meaning transfer model suggests, the symbolic meaning attributed to the endorsers, such as personality, lifestyle, and status can be transferred to the products being endorsed [9]. Furthermore, this meaning transfer process is determined by a match-up or fit between the endorsers and the endorsed offerings [13]. A high fit is found to produce better persuasive effects than a lack of fit does [13, 17]. Following this rationale, we propose that when the symbolic meaning associated with endorsers is consistent with the goal to follow normative appeals, consumers are more likely to respond positively to the pro-environmental advertisings.

Endorsers featuring high social status are aspirational referents that are admired and respected [5]. These endorsers represent the ideal self of the audience [14] who is motivated to enhance feelings of personal worth [29], which is similar to goals to follow injunctive norms [12]. Some previous research also points out that social prestige can activate the oughtness feelings for a norm [30]. Therefore, we propose that when high social status endorsers are paired with injunctive norm appeals, consumers are more likely to build up a connection between the endorsed pro-environmental behaviors and social status, and respond positively to the endorsement. Conversely, descriptive norm appeals serve the goals to behave efficiently and adaptively [11], which are, in some regards, inconsistent with the symbolic meaning ascribed to endorsers of high social status who provide high standards of social behaviors [31]. The work of Griskevicius, Tybur, and Van den Bergh [8] provides further evidence for this inconsistency. They demonstrate that the association between green consumption and social status will be mitigated when related costs are reduced, which suggests that a prevalent pro-environmental behavior as framed in descriptive norms may actually contradict the symbolic meaning represented by a high social status endorser. Therefore, we predict that the effectiveness of a high social status endorser would be mitigated when paired with descriptive norm appeals. In summary, we can hypothesize the following:

H1: When individuals with high social status endorse pro-environmental behaviors, injunctive norm appeals are more effective than descriptive norm appeals.

Previous literature suggests that a more average person image used in advertising can be viewed as a symbol representing consumers’ actual self [32], which, in our case, denotes people who try to maintain a positive impression with affordable costs. Some other research also points out that ordinary consumer endorsers can make the audience to believe that the endorsed activities can realistically be performed by them [33]. Therefore, common person endorsers, who play as exemplars for consumers pursuing the efficient/adaptive goal to follow descriptive norms [12], can strengthen the feeling that the costs associated with the endorsed pro-environmental behaviors are acceptable. In contrast, following injunctive norms to gain social approvals is more relevant to self-enhancement [29], which is not as consistent with the goals to motivate the actual self as descriptive norms are. Therefore, we can hypothesize the following:

H2: When ordinary consumers endorse pro-environmental behaviors, descriptive norm appeals are more effective than are injunctive norm appeals.

In the following section, we have conducted 3 experiments to provide evidences for the hypotheses. In Experiment 1, the effects of endorser social status and normative appeals were examined in a context of celebrity vs. ordinary consumer endorser. In Experiment 2, we extended the findings by directly manipulating the endorser social status with status labels. Experiment 3 replicated the previous two studies by manipulating social status with occupational roles, and with participants from ordinary citizens to provide robust evidence for the hypotheses.

## Experiment 1

In Experiment 1, we examine the hypotheses in a celebrity vs. ordinary consumer endorser context. As previous literature points out, celebrities are authority figures [7] who possess higher social status than do ordinary consumers [6], therefore, we try to explore whether this discrepancy in social status between celebrities and ordinary consumers would affect endorsement effectiveness when paired with different forms of normative appeals as predicted in the hypotheses.

### Design and measures

A 2 (endorser social status: celebrity vs. ordinary consumer) × 2 (normative appeals: injunctive vs. descriptive) between-subjects design was used in Experiment 1. The independent variables were manipulated through the design of advertising posters featuring an endorser and normative appeals to behave in an environmentally friendly manner. To select the celebrity endorser, we referred to the 2016 list of the most commercially valuable celebrities in First Financial Week (a prominent business information website in China), and the top five celebrities (Hu Ge, Angelababy, Deng Chao, Fan Bingbing, and Liu Tao) were selected. Twenty undergraduates in South Central China participated in the pretest to score the perceived credibility of the five selected celebrities after viewing their photos and stories (one 7-point semantic differential scale). Hu Ge, an actor with the highest average score of the five celebrities (M = 5.13), was then selected as the endorser for the celebrity endorsement condition.

Considering that participants in Experiment 1 were mainly from university, and to keep consistent with the gender of the chosen celebrity endorser, photos of 5 male university students were used as alternative ordinary consumer endorsers. Twenty undergraduates were invited to score these photos on perceived credibility (one 7-point semantic differential scale), and the one with the highest average score (M = 4.57) was selected as the ordinary consumer endorser.

Setting the temperature for air conditioning was chosen as the experiment background. China has established strict rules for setting air conditioning temperatures in public places: it should not be lower than 26°C in the summer, and it should not be higher than 20°C in the winter. However, consumers are not required to follow those rules in their private homes. In a brief pilot study, 13 of the 20 undergraduates surveyed indicated that they had not paid much attention to this issue. Therefore, the choice of this behavior suits the need for social norm research [12]. We referred to existing research to frame two types of normative appeals for pro-environmental behaviors [10, 12], and the injunctive and the descriptive norm appeals were expressed as follows: “every university student should save energy; please set the air conditioning temperature above 26°C in the summer and below 20°C in the winter” (injunctive norm) and “more and more university students set the air conditioning temperature above 26°C in the summer and below 20°C in the winter; let’s save energy together” (descriptive norm).

The endorsement effectiveness was examined by measuring attitudes towards the advertisements and intentions to act with scales adapted from prior literature [5]. The attitudes towards advertisements were measured with three 7-point Likert items: “I like this advertisement”, “This advertisement is appealing”, and “This advertisement gives me a good impression” (1 = strongly disagree, 7 = strongly agree; Cronbach’s α = .91). We averaged the scores to create an attitude scale. The intentions to act were measured by two 7-point Likert items: “I will pay attention to the setting temperature when I use air conditioning in the future” and “I am willing to remind other people to set the appropriate temperature for air conditioning” (1 = strongly disagree, 7 = strongly agree; Cronbach’s α = .76). We also averaged the scores to create an intention scale.

Because we used a real celebrity figure in experiment 1, this might produce a variation in endorsers’ attractiveness across different experiment conditions, thus confounding the results [14]; therefore, we measured the subjects’ evaluation of the endorsers’ attractiveness with three 7-point semantic differential items (bad/good, dislike/like, negative/positive, Cronbach’s α = .88), and average the scores to create an overall evaluation of endorser attractiveness to rule out this possibility.

As previous literature shows, people’s environmental knowledge plays a role in influencing their willingness to participate in pro-environmental activities [34, 35]. In order to eliminate such interference, we used three 7-point items (knowledge of the greenhouse effect, acid rain and haze; 1 = very unfamiliar, 7 = very familiar; Cronbach’s α = .72) adapted from previous research [35] and averaged them to measure the degree of participants’ subjective knowledge of related environmental issues.

### Participants and procedure

A total of 120 undergraduates (41 male, 79 female) in South Central China were invited to participate in this experiment for a souvenir worth ¥5. Four posters based on the theme mentioned above were created. Each poster included a photo of the endorser, statements about why one should set the air conditioning temperature, and the normative appeals. Participants were first briefed about the experiment procedure, and after providing their consent orally, they were arranged to randomly view one of the four posters. After viewing the poster, each participant was asked to write a brief sentence describing the theme of the poster and then completed the measurement scales anonymously. Six samples that did not describe the poster theme correctly were excluded, yielding 114 valid samples (39 male, 75 female).

### Data analysis

The attractiveness of endorsers may vary across experimental conditions due to the manipulation of endorser social status; we first applied a 2 (endorser social status) × 2 (normative appeals) analysis of variance (ANOVA) on endorser attractiveness to eliminate this potential confounding effect. The results showed that evaluations of the attractiveness of celebrity endorser were higher than that of the ordinary consumer (M_celebrity_ = 4.78, SD = .88; M_ordinary consumer_ = 4.39, SD = .71; F (1, 110) = 6.80, p = .01). Meanwhile, there was no significant difference in the endorser attractiveness evaluation between two normative appeal conditions (M_injunctive_ = 4.62, SD = .85; M_descriptive_ = 4.54, SD = .80; F (1, 110) = .35; p = .56); the interaction effect of endorser social status and normative appeals was not significant either (F (1, 110) = .005; p = .94). These findings could rule out the possibility that the attractiveness of endorsers affected their persuasiveness with different normative messages.

**Fig 1:**
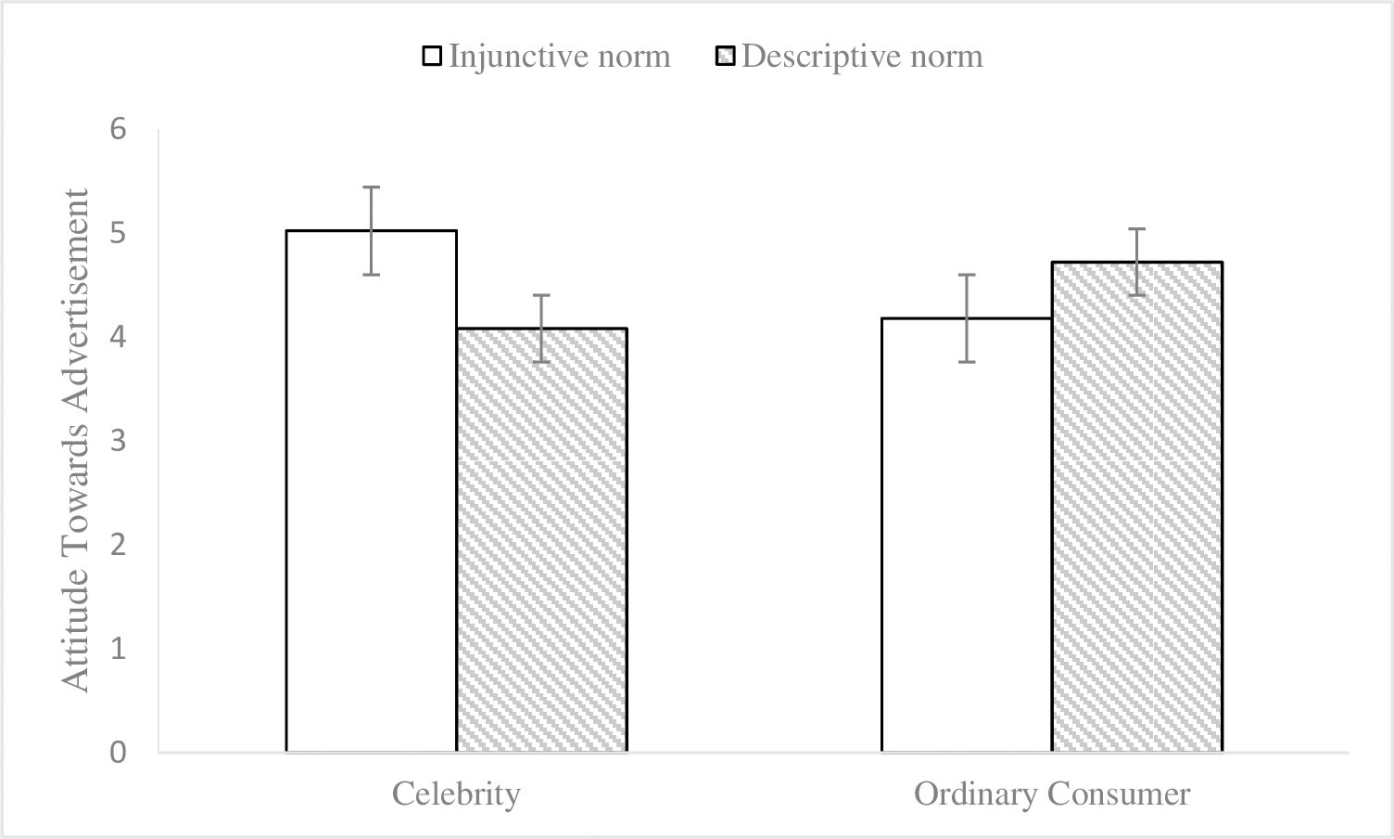
Interaction effect on attitude towards advertisement in Experiment 1. 2 (endorser social status: celebrity vs. ordinary consumer) × 2 (normative appeals: injunctive vs. descriptive) between-subjects design. 95% ci. N = 120.

**Fig 2:**
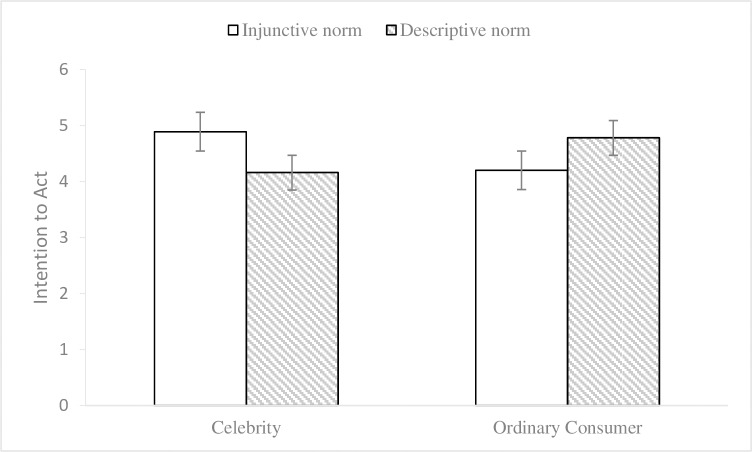
Interaction effect on intention to act in Experiment 1. 2 (endorser social status: celebrity vs. ordinary consumer) × 2 (normative appeals: injunctive vs. descriptive) between-subjects design. 95% ci. N = 120.

To control the potential effects of participants’ environmental knowledge and gender on the results [35, 36], we conducted a 2 (endorser social status) × 2 (normative appeals) multivariate analysis of covariance (MANCOVA, environmental knowledge and gender as covariates) on participants’ attitudes towards advertisements and intentions to act to examine endorsement effectiveness. The results showed that there were no significant main effects of the endorser social status on attitudes towards advertisements (M_celebrity_ = 4.56, SD = 1.00; M_ordinary consumer_ = 4.43, SD = .78; F (1, 108) = .30, p = .58, η2 p = .003) or on the intentions to act (M_celebrity_ = 4.54, SD = .88; M_ordinary consumer_ = 4.47, SD = .83; F (1, 108) = .003, p = .96, η2 p < .001). No significant main effects were found for social norm appeals on attitudes towards advertisements (M_injunctive_ = 4.59, SD = .91; M_descriptive_ = 4.39, SD = .88; F (1, 108) = 1.43, p = .23, η2 p = .013) or on the intentions to act (M_injunctive_ = 4.54, SD = .82; M_descriptive_ = 4.46, SD = .89; F (1, 108) = .12, p = .73, η2 p = .001). The interaction effect (see Figs 1 and 2) of endorser social status and social norm appeals was significant for attitudes towards advertisements (F (1, 108) = 21.36, p< .001, η2 p = .165) and intentions to act (F (1, 108) = 16.63, p < .001, η2 p = .133). Subsequently, the results of simple effects analyses showed that, when the endorser was a celebrity, the impacts of injunctive norm appeal on attitudes towards advertisements (M_injunctive_ = 5.02, SD = .90; M_descriptive_ = 4.08, SD = .87; F (1, 109) = 17.06, p < .001) and intentions to act (M_injunctive_ = 4.89, SD = .79; M_descriptive_ = 4.16, SD = .81; F (1, 109) = 9.89, p = .002) were better than those of the descriptive norm appeal. Meanwhile, when the endorser was an ordinary consumer, the effects of descriptive norm appeal on attitudes towards advertisements (M_injunctive_ = 4.18, SD = .70; M_descriptive_ = 4.72, SD = .77; F (1, 109) = 6.14, p = .015) and intentions to act (M_injunctive_ = 4.20, SD = .69; M_descriptive_ = 4.78, SD = .88; F (1, 109) = 7.18, p = .008) were better than those of the injunctive norm appeal. Thus, the results of the data analysis provided evidence for Hypotheses 1 and 2.

## Experiment 2

Experiment 1 offers a very unique setting to ascertain the interplay between endorser social status and normative appeals on the endorsement effectiveness. In Experiment 2, we provide more evidence for the hypotheses with a new method to manipulate social status. As previous literature suggests, displaying of prestige can serve as status symbols [37]; therefore, we try to directly manipulate endorser social status with status labels to provide further evidence for the hypotheses in Experiment 2.

### Design and procedure

A 2(endorser social status: excellent student vs. normal student) × 2 (normative appeals: injunctive vs. descriptive) between-subjects design was used in Experiment 2. We used photos of five female university students as alternative endorsers and asked 20 undergraduates to score the credibility of the endorsers after viewing these images. The image with the highest scores was selected as the endorser (M = 4.87). We used “nationally excellent university”, a title awarded by the Ministry of Education of China, to manipulate endorser social status. The chosen endorser was then labeled “nationally excellent university student, Chen Jia” in the high social status condition, whereas the label was just “university student, Chen Jia” in the ordinary consumer endorsement condition. Except for the endorsers, other procedures were the similar as in Experiment 1.

The measurements used in Experiment 1 were used here to measure attitudes towards advertisements (Cronbach’s α = .93) and intentions to act (Cronbach’s α = .88). In Experiment 2, we asked participants to evaluate the endorsers for social status to serve as manipulation checks (a 7-point semantic differential item). As prior literature suggests, social status could affect endorser trustworthiness and attractiveness [5, 7], which are predictors of endorsement effectiveness [14, 17]; therefore, we also asked participants to evaluate the endorsers for attractiveness and trustworthiness (each with one 7-point semantic differential item) to rule out the alternative explanations. In addition, we measured the environmental knowledge of the participants as control variables with scales used in Experiment 1 (Cronbach’s α = .79).

Eighty undergraduates from South China participated in Experiment 2 for course credit. They gave their consents to participate orally. We ultimately obtained 78 valid samples (28 male, 50 female) after excluding two participants that did not describe the poster theme appropriately.

### Data analysis

A 2 (endorser social status) × 2 (normative appeals) multivariate analysis of variance (MANOVA) was performed on participants’ evaluation of endorser social status, attractiveness, and trustworthiness to check the adequacy of our manipulation of endorser social status, and to rule out potential interferences of endorser trustworthiness and attractiveness. The results revealed a significant difference in the evaluation of endorsers social status based on different social labels (M_excellent_ = 5.05, SD = 1.12; M_ordinary_ = 4.15, SD = .71; F (1, 74) = 18.16, p < .001), neither the main effect of normative appeals (M_injunctive_ = 4.67, SD = 1.13; M_descriptive_ = 4.54, SD = .94; F (1, 74) = .25, p = .618) nor their interaction on endorser social status was significant (F (1, 74) = 3.73, p = .057), which showed that the social status of the endorsers was successfully manipulated. Furthermore, the excellent student were perceived to be more trustworthy than the ordinary student (M_excellent_ = 5.23, SD = .99; M_ordinary_ = 4.79, SD = .80; F (1, 74) = 4.45, p = .038); neither the main effect of normative appeals (M_injunctive_ = 5.18, SD = .91; M_descriptive_ = 4.85, SD = .90; F (1, 74) = 2.53, p = .116) nor their interaction on endorser trustworthiness was significant (F (1, 74) = .02, p = .902). The main effects of social status (M_excellent_ = 5.21, SD = 1.03; M_ordinary_ = 5.26, SD = .91; F (1, 74) = .08, p = .782) and normative appeals (M_injunctive_ = 5.41, SD = .99; M_descriptive_ = 5.05, SD = .92; F (1, 74) = 2.73, p = .103) on endorser attractiveness were not significant; their interaction on endorser attractiveness was not significant either (F (1, 74) = .58, p = .45). These results suggested that the endorser trustworthiness and attractiveness did not vary across different normative conditions; their potential interference on the research results could be eliminated.

**Fig 3:**
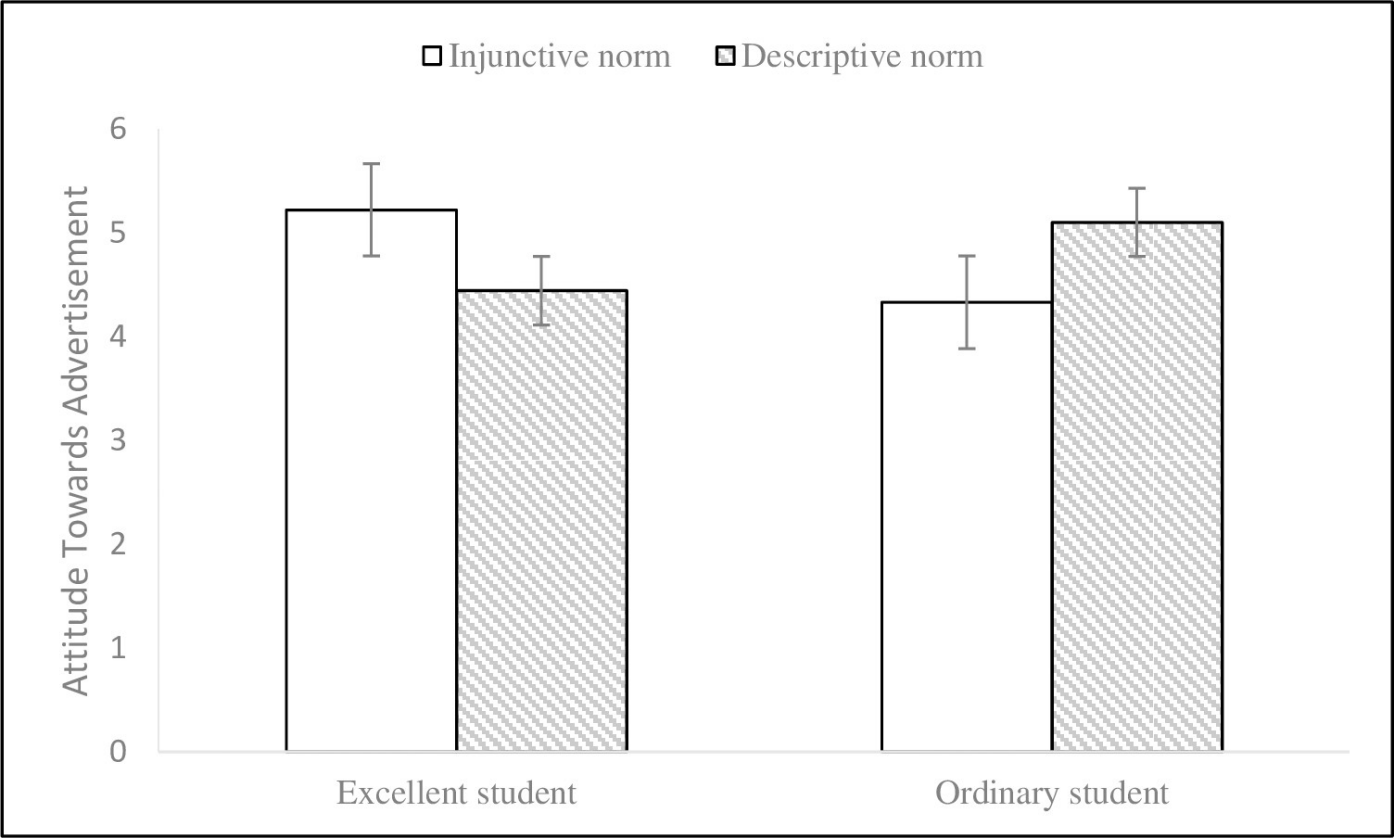
Interaction effect on attitude towards advertisement in Experiment 2. 2 (endorser social status: excellent student vs. normal student) × 2 (normative appeals: injunctive vs. descriptive) between-subjects design. 95% ci. N = 78.

**Fig 4:**
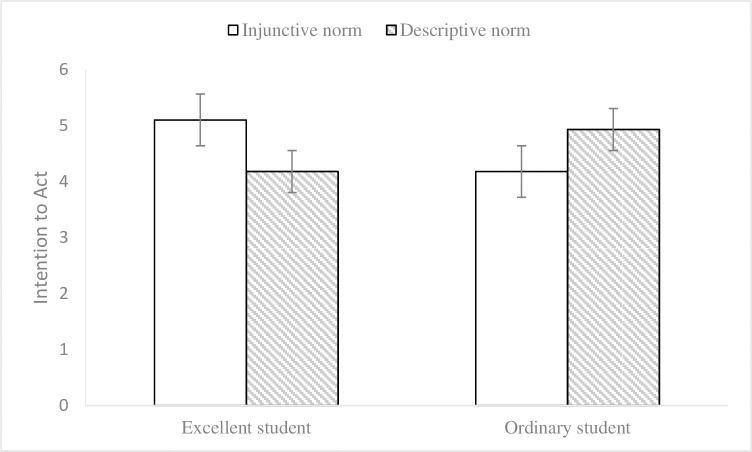
Interaction effect on intention to act in Experiment 2. 2 (endorser social status: excellent student vs. normal student) × 2 (normative appeals: injunctive vs. descriptive) between-subjects design. 95% ci. N = 78.

Subsequently, we adopted a 2 (endorser social status) × 2 (normative appeals) MANCOVA (environmental knowledge and gender as covariates) on participants’ attitudes towards advertisements and intentions to act to test the hypotheses. The results of data analysis (see Figs 3 and 4) showed that the effects of endorser social status on attitudes towards advertisements (M_excellent_ = 4.84, SD = 1.08; M_ordinary_ = 4.73, SD = 1.32; F (1, 72) = .26, p = .610, η2 p = .004) and intentions to act (M_excellent_ = 4.65, SD = 1.15; M_ordinary_ = 4.56, SD = 1.08; F (1, 72) = .49, p = .488, η2 p = .007) were not significant and that the effects of social norm appeals on attitudes towards advertisements (M_injunctive_ = 4.79, SD = 1.27; M_descriptive_ = 4.78, SD = 1.15; F (1, 72) = .01, p = .928, η2 p < .001) and intentions to act (M_injunctive_ = 4.65, SD = 1.28; M_descriptive_ = 4.56, SD = .92; F (1, 72) = .19, p = .664, η2 p = .003) were not significant either. In contrast, endorser social status and social norm appeals had significant interaction impacts on attitudes towards advertisements (F (1, 72) = 7.86, p = .006, η2 p = .098) and intentions to act (F (1, 72) = 11.43, p = .001, η2 p = .137). Simple effects analyses further illustrated that the impacts of injunctive norm appeal on attitudes towards advertisements (M_injunctive_ = 5.22, SD = 1.15; M_descriptive_ = 4.44, SD = .88; F (1, 73) = 4.33, p = .041) and intentions to act (M_injunctive_ = 5.10, SD = 1.25; M_descriptive_ = 4.18, SD = .82; F (1, 73) = 7.49, p = .008) were better when the endorser was labeled as an excellent student. Meanwhile, descriptive norm appeal produced more impacts on attitudes towards advertisements (M_injunctive_ = 4.33, SD = 1.26; M_descriptive_ = 5.10, SD = 1.30; F (1, 73) = 3.75, p = .057) and intentions to act (M_injunctive_ = 4.18, SD = 1.17; M_descriptive_ = 4.93, SD = .88; F (1, 73) = 4.41, p = .039) when an ordinary student label was provided for the endorser. Hypotheses 1 and 2 were again supported.

## Experiment 3

Participants in Experiment 1 and 2 are undergraduate students, which may limit the generalizability of our findings. In Experiment 3, we try to replicate our findings with samples of ordinary citizens. In addition, we refer to the work of Newton, Wong, and Newton [5] to manipulate endorser social status with occupational roles.

### Design and procedure

A 2(endorser social status: manager vs. receptionist) × 2 (normative appeals: injunctive vs. descriptive) between-subjects design was used in Experiment 3. In the high social status conditions, the endorser was described as a hotel manager, whereas in the low social status conditions the endorser was described as a hotel receptionist. We manipulated the normative appeals by varying the text in the advertisement as we did in Experiment 1 and 2.

Compared to participants in Experiment 1 and 2, participants in Experiment 3 are ordinary citizens with diverse social backgrounds, which demands a larger sample size than we used before. Therefore, four hundred residents aged 19 to 58 from a neighborhood community in South China were invited and completed a paper-and-pencil version of the questionnaire for a coupon worth ¥5. As in previous experiments, their consents were given orally. They were assigned randomly to view one of the four posters, and then reported their attitudes towards advertisements (Cronbach’s α = .942) and intentions to act (Cronbach’s α = .886) on the same scales as used in the prior experiments. Participants were also asked to score their evaluation of the endorser’s social status, trustworthiness and attractiveness, as well as their knowledge about related environmental issues (Cronbach’s α = .891). As in experiment 1 and 2, we have excluded 69 participants who did not describe the poster theme correctly. Finally, we have got 331 participants for Experiment 3 (177 male, 154 female).

### Data analysis

We first applied a 2 (endorser social status) × 2 (normative appeals) MANOVA on endorser social status, trustworthiness and attractiveness. The results confirmed a significant difference in the perception of endorsers’ social status based on different occupational roles (M_manager_ = 5.37, SD = 1.09; M_receptionist_ = 3.53, SD = 1.02; F (1, 327) = 250.54, p < .001); the perceptions of endorser social status did not differ between different norm appeal conditions (M_injunctive_ = 4.49, SD = 1.43; M_descriptive_ = 4.41, SD = 1.37; F (1, 327) = .54, p = .462); and their interaction was not significant (F (1, 327) = .23, p = .632). Therefore, the manipulation of social status was successful. In addition, endorsers with higher social status were perceived to be more trustworthy (M_manager_ = 4.92, SD = 1.12; M_receptionist_ = 4.68, SD = 1.10; F (1, 327) = 4.07, p = .044), but not more attractive (M_manager_ = 4.51, SD = 1.16; M_receptionist_ = 4.35, SD = 1.12; F (1, 327) = 1.64, p = .201); no significant differences in endorser trustworthiness (M_injunctive_ = 4.83, SD = 1.15; M_descriptive_ = 4.77, SD = 1.08; F (1, 327) = .28, p = .599) and attractiveness (M_injunctive_ = 4.47, SD = 1.18; M_descriptive_ = 4.40, SD = 1.10; F (1, 327) = .34, p = .561) were found between the two normative appeal conditions; the interactions on endorser trustworthiness (F (1, 327) = 1.74, p = .189) and attractiveness (F (1, 327) = .01, p = .925) were not significant. These results provided evidence that the trustworthiness and attractiveness of endorsers could not affect the research results.

**Fig 5:**
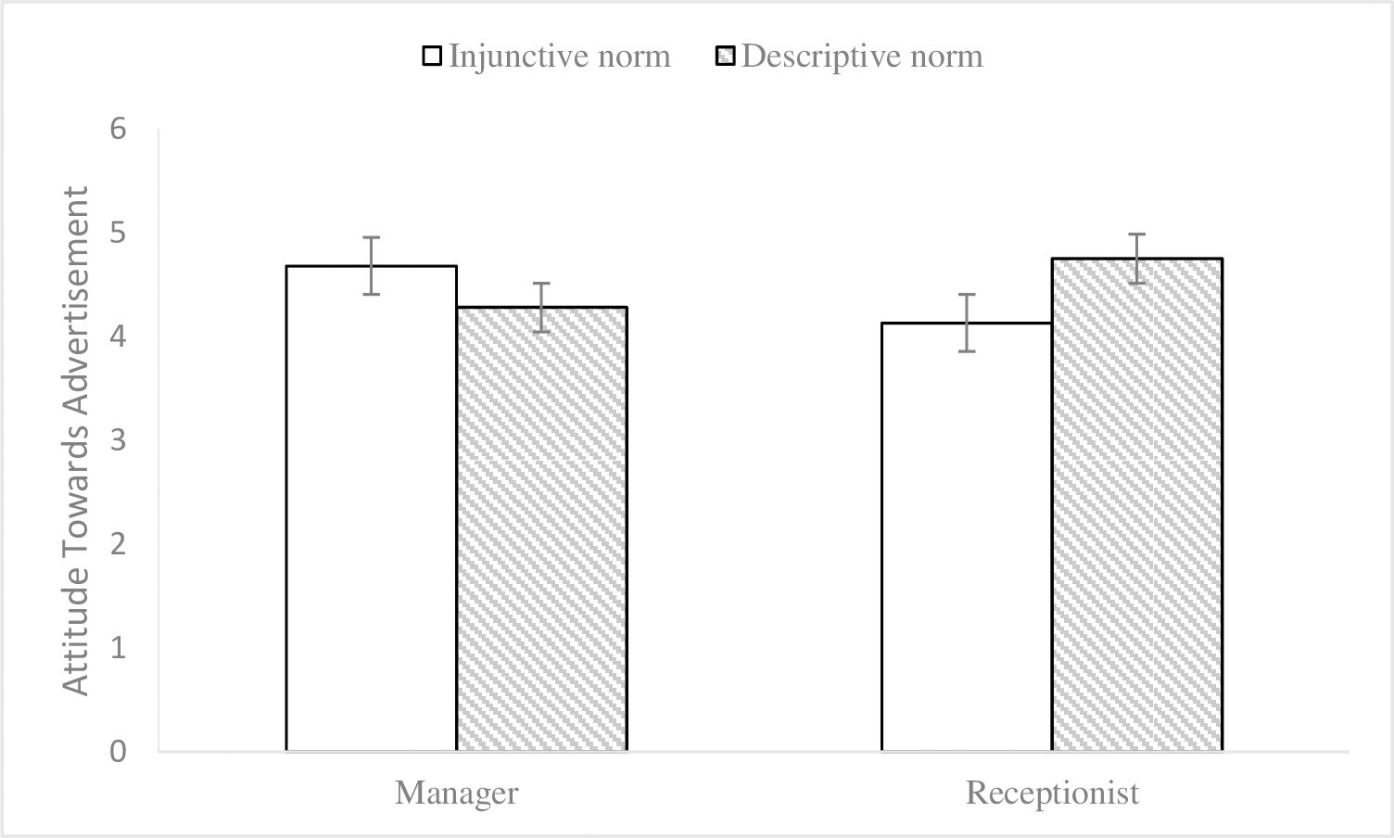
Interaction effect on attitude towards advertisement in Experiment 3. 2 (endorser social status: manager vs. receptionist) × 2 (normative appeals: injunctive vs. descriptive) between-subjects design. 95% ci. N = 331.

**Fig 6:**
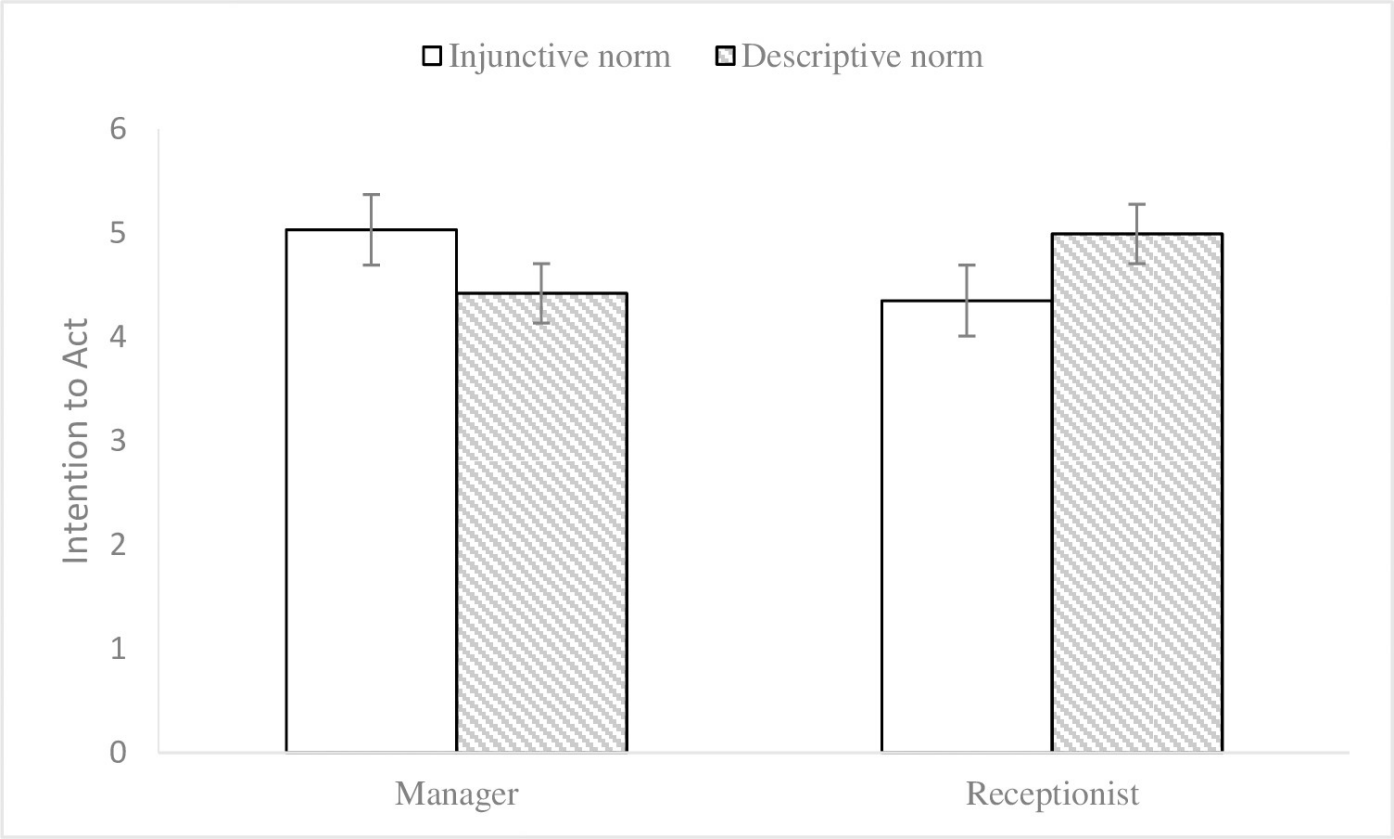
Interaction effect on intention to act in Experiment 2. 2 (endorser social status: manager vs. receptionist) × 2 (normative appeals: injunctive vs. descriptive) between-subjects design. 95% ci. N = 331.

Subsequently, a 2(endorser social status) × 2(normative appeals) MANCOVA (environmental knowledge, gender, and age as covariates) on participants’ attitudes towards advertisements and intentions to act was performed. The results of data analysis (see Figs 5 and 6) showed that the effects of endorser social status on attitudes towards advertisements (M_manager_ = 4.47, SD = 1.05; M_receptionist_ = 4.45, SD = 1.14; F (1, 324) = .41, p = .525, η2 p = .001) and intentions to act (M_manager_ = 4.72, SD = 1.19; M_receptionist_ = 4.68, SD = 1.25; F (1, 324) = .83, p = .362, η2 p = .003) were not significant and that the effects of social norm appeals on attitudes towards advertisements (M_injunctive_ = 4.40, SD = 1.12; M_descriptive_ = 4.51, SD = 1.07; F (1, 324) = 1.26, p = .263, η2 p = .004) and intentions to act (M_injunctive_ = 4.69, SD = 1.22; M_descriptive_ = 4.71, SD = 1.21; F (1, 324) = .04, p = .844, η2 p < .001) were not significant either. In contrast, endorser social status and social norm appeals had significant interaction impacts on attitudes towards advertisements (F (1, 324) = 27.00, p < .001, η2 p = .077) and intentions to act (F (1, 324) = 38.68, p < .001, η2 p = .107). Simple effects analyses further illustrated that the impacts of injunctive norm appeal on attitudes towards advertisements (M _injunctive_ = 4.68, SD = 1.06; M_descriptive_ = 4.28, SD = 1.01; F (1, 325) = 8.28, p = .004) and intentions to act (M_injunctive_ = 5.03, SD = 1.07; M_descriptive_ = 4.42, SD = 1.23; F (1, 325) = 18.07, p < .001) were better when the endorser was described as a manager. Meanwhile, descriptive norm appeal produced more impacts on attitudes towards advertisements (M_injunctive_ = 4.13, SD = 1.11; M_descriptive_ = 4.75, SD = 1.09; F (1, 325) = 19.87, p < .001) and intentions to act (M _injunctive_ = 4.35, SD = 1.28; M_descriptive_ = 4.99, SD = 1.13; F (1, 325) = 20.44, p < .001) when the endorser was described as a receptionist. These results provided further evidence for hypotheses 1 and 2.

## General discussion

The current research examines how the endorser social status affects endorsement effectiveness for pro-environmental behaviors in combination with different types of normative appeals. In 3 experiments, we find that high social status endorsers are more effective in influencing pro-environmental behaviors when paired with injunctive norm appeals than with descriptive norm appeals; in contrast, ordinary consumer endorsers predict more positive pro-environmental attitudes and intentions in combination with descriptive norm appeals than with injunctive norm appeals. This finding is true when the endorser is a real public figure with high social status (experiment 1), endowed with status labels (experiment 2) or occupational roles (experiment 3) indicating social status or prestige.

This research makes several contributions to existing research. First, we have outlined a framework to understand endorsement effectiveness in the context of environmental protection. As prior works suggest, while high social status endorsers are more effective for status-related products [5, 23, 24], ordinary consumer endorsers have an advantage in enhancing consumers’ perceived efficacy [32]. We build on these findings by highlighting the role that frames of normative appeals can play in motivating consumers to protect the environment [11]. By doing this, we demonstrate a match-up effect between endorser social status and normative appeals. That is, when the symbolic meaning of endorser social status is consistent with the distinct goal that motivates consumers to follow different types of social norms, consumers will respond more favorably. Furthermore, our findings indicate that pro-environmental behaviors are more likely to be associated with social status when it is framed as social obligation. This result helps us to deepen the knowledge about the role social status can play in endorsement effectiveness by offering a new boundary condition.

Second, our findings have some implications for social norm research as well. Our work shows that consumers will respond more favorably to the normative appeal when it is endorsed by an appropriate message endorser. This result extends prior literature by offering an integrated framework to analyze effects of reference groups on norm-consistent behaviors. For example, Lindenberg, Joly, and Stapel [30] have found that social prestige could arouse people’s perception of social obligations and engage them to follow injunctive norms, whereas in Goldstein, Cialdini, and Griskevicius’ work [28], the descriptive norm of guests in the same room are more influential than that of groups with whom they are more identified. Our findings provide additional evidence for these findings and suggest that the fundamentally distinct goals related to injunctive and descriptive norms [12] may account for the differential effects of reference groups.

At last, our findings also contribute to the literature concerning effectiveness of celebrity endorser. In Experiment 1, our hypotheses were tested in a context of celebrity vs. ordinary consumer endorser; the findings provide more evidence to the notion that status concerns may be a critical boundary condition for the effectiveness of celebrity endorser. For example, Kamins [23] finds that celebrity endorsers are more appropriate than noncelebrity endorsers for products with a high social or psychological risk; Winterich, Gangwar, and Grewal [7] find that celebrity endorsers are more effective for audiences who are high in power distance beliefs. Echoing this stream of literature, our findings suggest that the effectiveness of celebrity endorsers for pro-environmental behaviors will be more prominent when the oughtness facet of the behavior is stressed. This helps to deepen our knowledge about how to utilize celebrities in advertising more effectively.

Our findings also have great implications for the practice of green advertising. Environment protection is more than an issue of economic concern [38], and encouraging more consumers to adopt an environmentally friendly lifestyle is a great challenge for government and pro-environmental organizations [10]. Since both high social status endorsers and ordinary consumer endorsers have some merits and are widely used in pro-environmental advertising, it is of great importance to know how to strengthen their effectiveness accordingly. As our findings indicate, marketers should pay attention to aligning the normative message with the endorser’s social status. Specifically, when endorsers with high social status (e.g., celebrities or status labels) are used, advertisers can employ an injunctive norm to stress the social obligation to act in an environmentally friendly manner. When an ordinary consumer is used as message endorser, a descriptive norm to highlight the prevalence of the endorsed behaviors may be a better choice.

## Limitations and future research

One limitation of our research stems from the specific behavioral domain we chose. The pilot study in Experiment 1 showed that participants were not highly involved with this behavior. However, prior literature points out that the degree of consumer involvement may influence their perception of and willingness to follow social norms [34, 39], which was not considered in our experiments, so the generalizability of these effects in other behavior contexts still should be examined in the future research. Another limitation of our research is that we only measured the respondents’ intention to follow the social norms. Though prior literature demonstrates that behavior intentions and actual behaviors are highly relevant [5], a field study that employs actual measures of behavior is welcomed in the future studies.

Our work also provides some potentially fruitful avenues for future research. First, we have examined the interplay between the endorser social status and normative appeals in the context of pro-environmental behaviors, which lead consumers to a trade-off between self-sacrifice and social benefits [10]. However, it is likely that a similar interaction would be observed in commercial advertising contexts, especially for products that are status-related. In future research, we could test our findings in other product settings. Second, in Experiment 1, our findings suggested no main effect of the endorser type on the endorsement effectiveness, which may indicate that celebrity endorsers are not always superior to ordinary consumer endorsers. In the future research, we could further examine when and how this effect will happen. Third, this research was conducted in mainland China, which is known as a collectivist culture [26]. Previous research suggests that cultural background might alter the relative effectiveness of injunctive and descriptive norms [34]. Thus, future research could investigate whether a similar result would be obtained in individualist cultures.

## Supporting information

S1 File Data of Experiment 1. (XLS)

S2 File Data of Experiment 2. (XLS)

S3 File Data of Experiment 3. (XLS)

S4 File Experimental materials for Experiment 1, 2, and 3. (DOCX)

S5 File Detailed results of data analysis. (DOCX)
