# Supplementary material for: The interplay between endorser social status and normative appeals on the endorsement effectiveness of pro-environmental behaviors
Source: PLoS One. 2019 Jan 15;14(1):e0210699. doi: 10.1371/journal.pone.0210699 (PMC6333400; doi:10.1371/journal.pone.0210699)

Materials and Wording of Experiment 1 (Faces are not covered in the experiment)

High status endorser – Injunctive norms


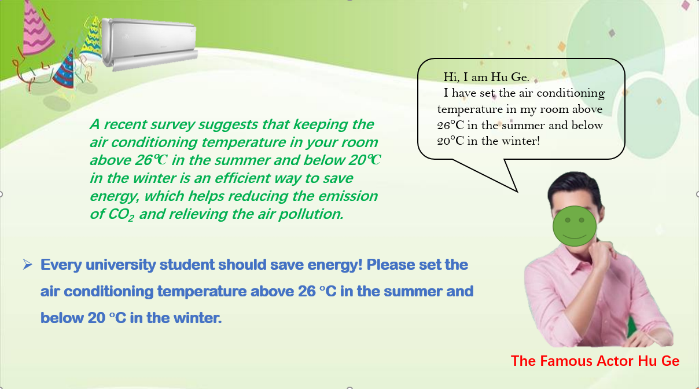


Ordinary consumer endorser - Injunctive norms


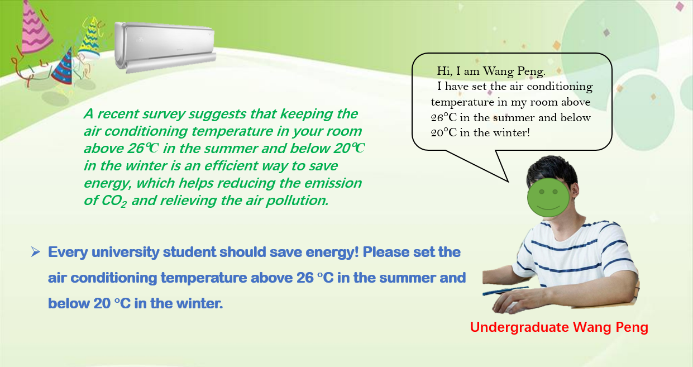


High status endorser – Descriptive norms


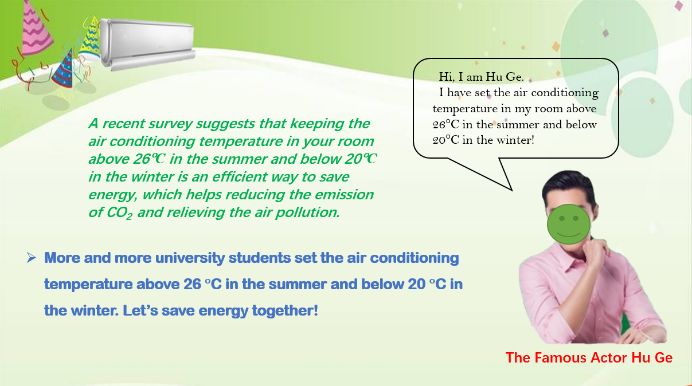


Ordinary consumer endorser - Descriptive norms


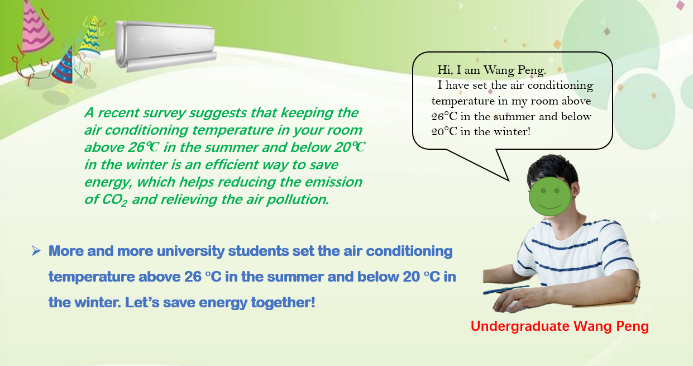


Materials and Wording of Experiment 2 (Faces are not covered in the experiment)

High status endorser – Injunctive norms


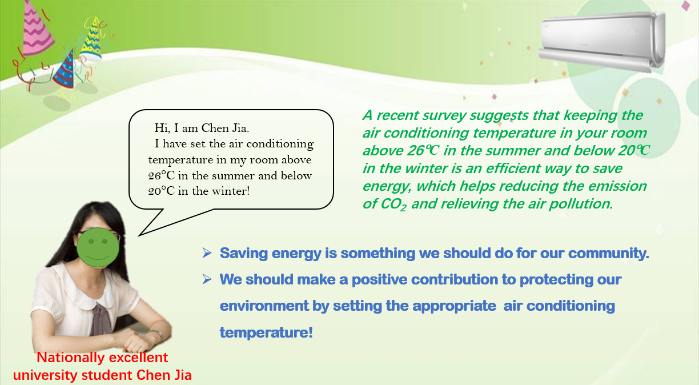


Ordinary consumer endorser - Injunctive norms


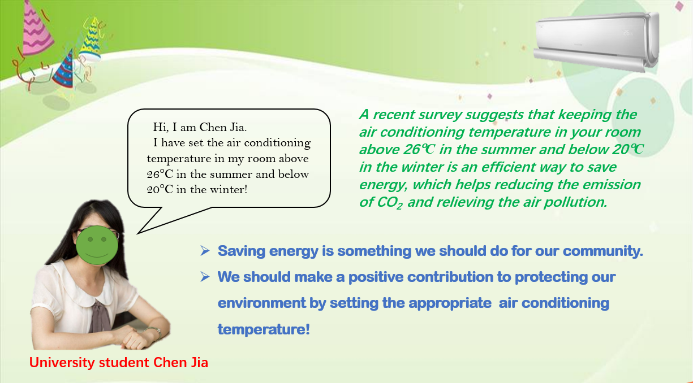


High status endorser – Descriptive norms


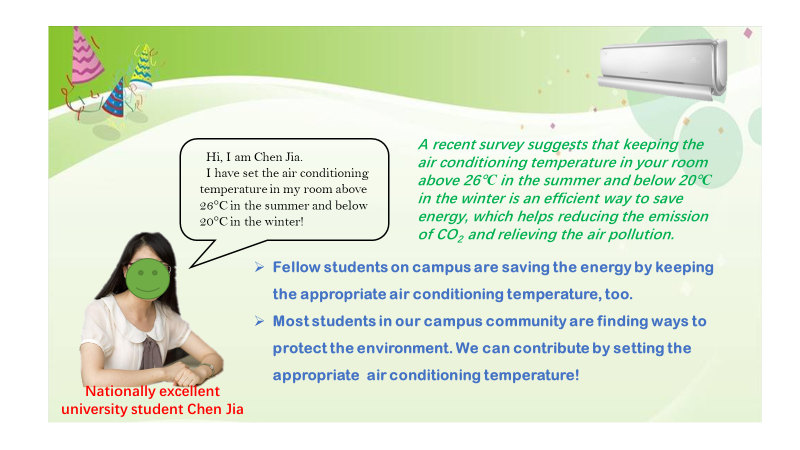


Ordinary consumer endorser - Descriptive norms


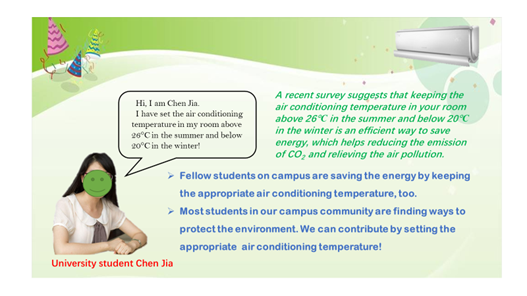


Materials and Wording of Experiment 3 (Faces are not covered in the experiment)

High status endorser – Injunctive norms


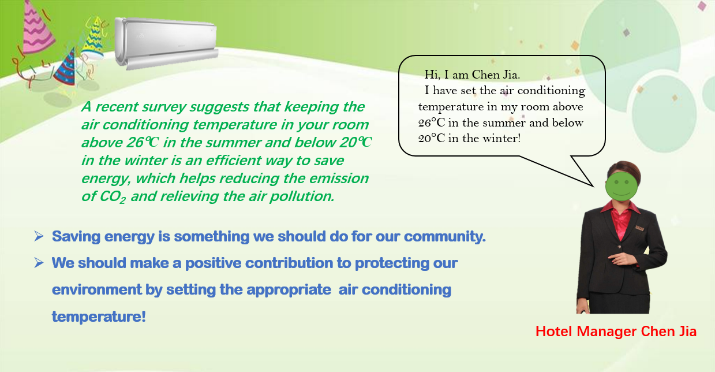


Ordinary consumer endorser - Injunctive norms


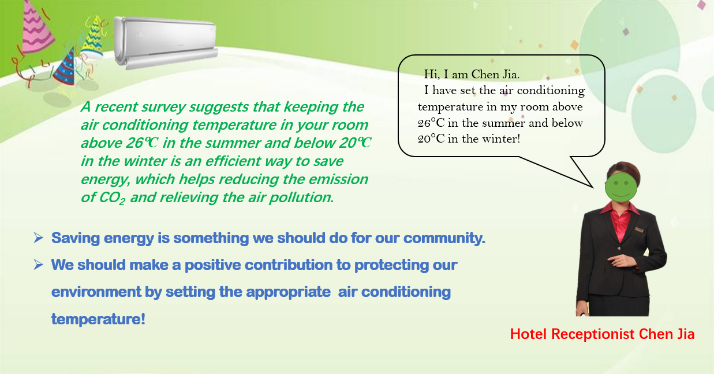


High status endorser – Descriptive norms


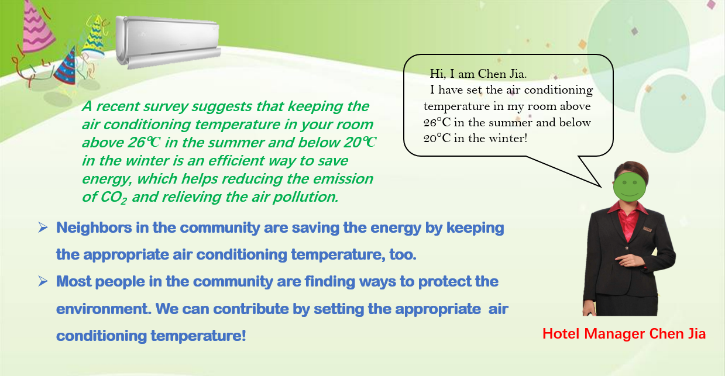


Ordinary consumer endorser - Descriptive norms


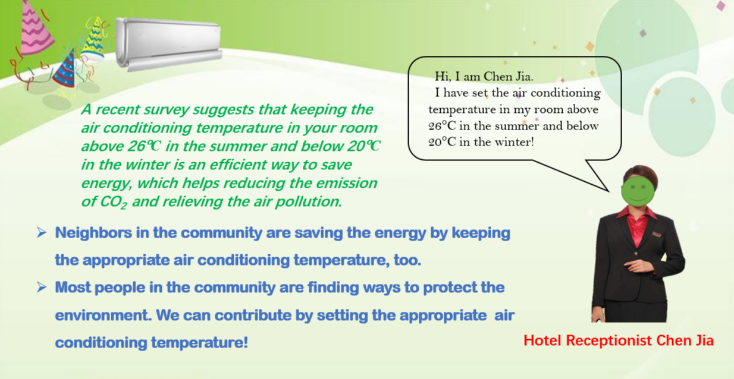

Supplement: S4 File — (DOCX) [file pone.0210699.s004.docx]
