# Supplementary material for: The interplay between endorser social status and normative appeals on the endorsement effectiveness of pro-environmental behaviors
Source: PLoS One. 2019 Jan 15;14(1):e0210699. doi: 10.1371/journal.pone.0210699 (PMC6333400; doi:10.1371/journal.pone.0210699)
Supplement: S5 File — (DOCX) [file pone.0210699.s005.docx]

Experiment 1

Manipulation and confounding check

Table 1 Descriptive statistics

| DV | Endorser | Social norm appeal | |  |
| --- | --- | --- | --- | --- |
|  |  | Injunctive | Descriptive | Sum |
| Attractive-ness | Celebrity | 4.82 (SD= .93，N=29)  (95% CI [4.46, 5.17]) | 4.74 (SD= .84，N=28)  (95% CI [4.41, 5.06]) | 4.78 (SD= .88) |
|  | Ordinary consumer | 4.43 (SD= .72，N=30 )  (95% CI [4.16, 4.70]) | 4.33 (SD=0.71，N=27 )  (95% CI [4.05, 4.61] ) | 4.39 (SD= .71) |
|  | Sum | 4.62 (SD= .85) | 4.54 (SD= .80) |  |

Table 2 Results of ANOVA

| Variables | df | F | Sig. | η2 p |
| --- | --- | --- | --- | --- |
| Endorser (E) | 1 | 6.80 | .01 | .058 |
| Social norm appeal (S) | 1 | .35 | .56 | .003 |
| E × S | 1 | .005 | .94 | .000 |
| Errors | 110 |  |  |  |

Hypotheses tests

Table 3 Descriptive statistics

| DV | Endorser | Social norm appeal | |  |
| --- | --- | --- | --- | --- |
|  |  | Injunctive | Descriptive | Sum |
| Attitude towards advertise-ment | Celebrity | 5.02 (SD= .90，N=29)  (95% CI [4.68, 5.37]) | 4.08 (SD= .87，N=28)  (95% CI [3.75, 4.42]) | 4.56 (SD=1.00) |
|  | Ordinary consumer | 4.18 (SD= .70，N=30 )  (95% CI [3.91, 4.44]) | 4.72 (SD=0.77，N=27 )  (95% CI [4.41, 5.02] ) | 4.43 (SD= .78) |
|  | Sum | 4.59 (SD= .91) | 4.39 (SD= .88) |  |
| Intention to act | Celebrity | 4.90 (SD= .79，N=29)  (95% CI [4.59, 5.20]) | 4.16 (SD= .81，N=28)  (95% CI [3.85, 4.47]) | 4.54 (SD= .88) |
|  | Ordinary consumer | 4.20 (SD= .69，N=30 )  (95% CI [3.94, 4.46]) | 4.78 (SD=0.88，N=27 )  (95% CI [4.43, 5.13] ) | 4.47 (SD= .83) |
|  | Sum | 4.54 (SD= .82) | 4.46 (SD= .89) |  |

Table 4 Results of MANCOVA

| DVs | IDVs | df | F | Sig. | η2 p |
| --- | --- | --- | --- | --- | --- |
| Attitude towards advertisement | Gender | 1 | 6.03 | .016 | .053 |
|  | Environmental Knowledge | 1 | .149 | .700 | .001 |
|  | Endorser (E) | 1 | .302 | .584 | .003 |
|  | Social norm appeal (S) | 1 | 1.434 | .234 | .013 |
|  | E × S | 1 | 21.360 | .000 | .165 |
|  | Errors | 108 |  |  |  |
| Intention to act | Gender | 1 | 8.523 | .004 | .073 |
|  | Environmental Knowledge | 1 | 4.318 | .040 | .038 |
|  | Endorser (E) | 1 | .003 | .958 | .000 |
|  | Social norm appeal (S) | 1 | .118 | .732 | .001 |
|  | E × S | 1 | 16.632 | .000 | .133 |
|  | Errors | 108 |  |  |  |

Experiment 2

Manipulation and confounding check

Table 5 Descriptive statistics

| DV | Endorser | Social norm appeal | |  |
| --- | --- | --- | --- | --- |
|  |  | Injunctive | Descriptive | Sum |
| Attractive-ness | Excellent  student | 5.30 (SD=1.08，N=20)  (95% CI [4.79, 5.81]) | 5.11 (SD= .99，N=19)  (95% CI [4.63, 5.58]) | 5.21 (SD=1.03) |
|  | Ordinary  student | 5.53 (SD= .91，N=19 )  (95% CI [5.09, 5.96 ]) | 5.00 (SD= .86，N=20 )  (95% CI [4.60, 5.40] ) | 5.26 (SD= .91) |
|  | Sum | 5.41 (SD= .99) | 5.05 (SD= .92) |  |
| Trustwort-hiness | Excellent  student | 5.40 (SD= .94，N=20)  (95% CI [4.96, 5.84]) | 5.05 (SD=1.02，N=19)  (95% CI [4.56, 5.55]) | 5.23 (SD= .99) |
|  | Ordinary  student | 4.95 (SD= .85，N=19 )  (95% CI [4.54, 5.36]) | 4.65 (SD=0.75，N=20 )  (95% CI [4.30, 5.00] ) | 4.79 (SD= .80) |
|  | Sum | 5.18 (SD= .91) | 4.85 (SD= .90) |  |
| Status | Excellent  student | 5.30 (SD=1.13，N=20)  (95% CI [4.77, 5.83]) | 4.79 (SD=1.08，N=19)  (95% CI [4.27, 5.31]) | 5.05 (SD=1.12) |
|  | Ordinary  student | 4.00 (SD= .67，N=19 )  (95% CI [3.68, 4.32]) | 4.30 (SD= .73，N=20 )  (95% CI [3.96, 4.64] ) | 4.15 (SD= .71) |
|  | Sum | 4.67 (SD=1.13) | 4.54 (SD= .94) |  |

Table 6 Results of MANOVA

| DVs | IDVs | df | F | Sig. | η2 p |
| --- | --- | --- | --- | --- | --- |
| Attractiveness | Endorser (E) | 1 | .077 | .782 | .001 |
|  | Social norm appeal (S) | 1 | 2.727 | .103 | .036 |
|  | E × S | 1 | .577 | .450 | .008 |
|  | Errors | 74 |  |  |  |
| Trustworthiness | Endorser (E) | 1 | 4.451 | .038 | .057 |
|  | Social norm appeal (S) | 1 | 2.530 | .116 | .033 |
|  | E × S | 1 | .015 | .902 | .000 |
|  | Errors | 74 |  |  |  |
| Status | Endorser (E) | 1 | 18.164 | .000 | .197 |
|  | Social norm appeal (S) | 1 | .251 | .618 | .003 |
|  | E × S | 1 | 3.726 | .057 | .048 |
|  | Errors | 74 |  |  |  |

Hypotheses tests

Table 7 Descriptive statistics

| DV | Endorser | Social norm appeal | |  |
| --- | --- | --- | --- | --- |
|  |  | Injunctive | Descriptive | Sum |
| Attitude towards advertise-ment | Excellent  student | 5.22 (SD=1.15，N=20)  (95% CI [4.68, 5.75]) | 4.44 (SD= .88，N=19)  (95% CI [4.02, 4.86]) | 4. 84 (SD=1.08) |
|  | Ordinary  student | 4.33 (SD=1.26，N=19 )  (95% CI [3.73, 4.94]) | 5.10 (SD=1.30，N=20 )  (95% CI [4.49, 5.71]) | 4.73 (SD=1.32) |
|  | Sum | 4.79 (SD=1.27) | 4.78 (SD=1.15) |  |
| Intention to act | Excellent  student | 5.10 (SD=1.25，N=20)  (95% CI [4.51, 5.69]) | 4.18 (SD= .82，N=19)  (95% CI [3.79, 4.58]) | 4.65 (SD=1.15) |
|  | Ordinary  student | 4.18 (SD=1.17，N=19 )  (95% CI [3.62, 4.75]) | 4.93 (SD=0.88，N=20 )  (95% CI [4.51, 5.34]) | 4.56 (SD=1.08) |
|  | Sum | 4.65 (SD=1.28) | 4.56 (SD= .92) |  |

Table 8 Results of MANCOVA

| DVs | IDVs | df | F | Sig. | η2 p |
| --- | --- | --- | --- | --- | --- |
| Attitude towards advertisement | Gender | 1 | 1.630 | .206 | .022 |
|  | Environmental Knowledge | 1 | 12.222 | .001 | .145 |
|  | Endorser (E) | 1 | .263 | .610 | .004 |
|  | Social norm appeal (S) | 1 | .008 | .928 | .000 |
|  | E × S | 1 | 7.861 | .006 | .098 |
|  | Errors | 72 |  |  |  |
| Intention to act | Gender | 1 | .068 | .795 | .001 |
|  | Environmental Knowledge | 1 | 27.916 | .000 | .279 |
|  | Endorser (E) | 1 | .486 | .488 | .007 |
|  | Social norm appeal (S) | 1 | .190 | .664 | .003 |
|  | E × S | 1 | 11.432 | .001 | .137 |
|  | Errors | 72 |  |  |  |

Experiment 3

Manipulation and confounding check

Table 9 Descriptive statistics

| DV | Endorser | Social norm appeal | |  |
| --- | --- | --- | --- | --- |
|  |  | Injunctive | Descriptive | Sum |
| Attractive-ness | Manager | 4.56 (SD=1.14，N=81)  (95% CI [4.30, 4.81]) | 4.47 (SD=1.18，N=85)  (95% CI [4.22, 4.73]) | 4.51 (SD=1.16) |
|  | Receptio-nist | 4.38 (SD=1.22，N=81 )  (95% CI [4.11, 4.65]) | 4.32 (SD=1.02，N=84)  (95% CI [4.10,4.54]) | 4.35 (SD=1.12) |
|  | Sum | 4.47 (SD=1.18) | 4.40 (SD=1.10) |  |
| Trustwort-hiness | Manager | 5.04 (SD=1.08，N=81)  (95% CI [4.80, 5.28]) | 4.81 (SD=1.15，N=85)  (95% CI [4.56, 5.06]) | 4.92 (SD=1.12) |
|  | Receptio-nist | 4.63 (SD=1.20，N=81 )  (95% CI [4.36, 4.89) | 4.73 (SD=1.01，N=84)  (95% CI [4.51,4.95]) | 4.68 (SD=1.10) |
|  | Sum | 4.83 (SD=1.15) | 4.77 (SD=1.08) |  |
| Status | Manager | 5.38 (SD=1.16，N=81)  (95% CI [5.13, 5.64]) | 5.35 (SD=1.02，N=85)  (95% CI [5.13, 5.57]) | 5.37 (SD=1.09) |
|  | Receptio-nist | 3.60 (SD=1.08，N=81 )  (95% CI [3.37, 3.84]) | 3.4 (SD= .95，N=84 )  (95% CI [3.26, 3.67]) | 3.53 (SD=1.02) |
|  | Sum | 4.49 (SD=1.43) | 4.41 (SD=1.37) |  |

Table 10 Results of MANOVA

| DVs | IDVs | df | F | Sig. | η2 p |
| --- | --- | --- | --- | --- | --- |
| Attractiveness | Endorser (E) | 1 | 1.643 | .201 | .005 |
|  | Social norm appeal (S) | 1 | .339 | .561 | .001 |
|  | E × S | 1 | .009 | .925 | .000 |
|  | Errors | 327 |  |  |  |
| Trustworthiness | Endorser (E) | 1 | 4.073 | .044 | .012 |
|  | Social norm appeal (S) | 1 | .278 | .599 | .001 |
|  | E × S | 1 | 1.736 | .189 | .005 |
|  | Errors | 327 |  |  |  |
| Status | Endorser (E) | 1 | 250.539 | .000 | .434 |
|  | Social norm appeal (S) | 1 | .541 | .462 | .002 |
|  | E × S | 1 | .229 | .632 | .001 |
|  | Errors | 327 |  |  |  |

Hypotheses tests

Table 11 Descriptive statistics

| DV | Endorser | Social norm appeal | |  |
| --- | --- | --- | --- | --- |
|  |  | Injunctive | Descriptive | Sum |
| Attitude towards advertise-ment | Manager | 4.68 (SD=1.06，N=81)  (95% CI [4.45, 4.91]) | 4.28 (SD=1.01，N=85)  (95% CI [4.06, 4.50]) | 4. 47 (SD=1.05) |
|  | Receptio-nist | 4.13 (SD=1.11，N=81 )  (95% CI [3.88, 4.37]) | 4.75(SD=1.09，N=84 )  (95% CI [4.52, 4.99]) | 4.45 (SD=1.14) |
|  | Sum | 4.40 (SD=1.12) | 4.51 (SD=1.07) |  |
| Intention to act | Manager | 5.03 (SD=1.07，N=81)  (95% CI [4.79, 5.27]) | 4.42 (SD=1.23，N=85)  (95% CI [4.16, 4.69]) | 4.72 (SD=1.19) |
|  | Receptio-nist | 4.35 (SD=1.28，N=81)  (95% CI [4.06, 4.63]) | 4.99 (SD=1.13，N=84 )  (95% CI [4.75, 5.24]) | 4.68 (SD=1.25) |
|  | Sum | 4.69 (SD=1.22) | 4.71 (SD=1.21) |  |

Table 12 Results of MANCOVA

| DVs | IDVs | df | F | Sig. | η2 p |
| --- | --- | --- | --- | --- | --- |
| Attitude towards advertisement | Age | 1 | 3.754 | .054 | .011 |
|  | Gender | 1 | .089 | .766 | .000 |
|  | Environmental Knowledge | 1 | 92.497 | .000 | .222 |
|  | Endorser (E) | 1 | .405 | .525 | .001 |
|  | Social norm appeal (S) | 1 | 1.257 | .263 | .004 |
|  | E × S | 1 | 26.996 | .000 | .077 |
|  | Errors | 324 |  |  |  |
| Intention to act | Age | 1 | 1.738 | .188 | .005 |
|  | Gender | 1 | 1.010 | .316 | .003 |
|  | Environmental Knowledge | 1 | 172.418 | .000 | .347 |
|  | Endorser (E) | 1 | .833 | .362 | .003 |
|  | Social norm appeal (S) | 1 | .039 | .844 | .000 |
|  | E × S | 1 | 38.682 | .000 | .107 |
|  | Errors | 324 |  |  |  |
